# Supplementary figures and images for: A nonsense mutant of the hepatitis B virus large S protein antagonizes multiple tumor suppressor pathways through c-Jun activation domain-binding protein1
Source: PLoS One. 2019 Mar 14;14(3):e0208665. doi: 10.1371/journal.pone.0208665 (PMC6417713; doi:10.1371/journal.pone.0208665)

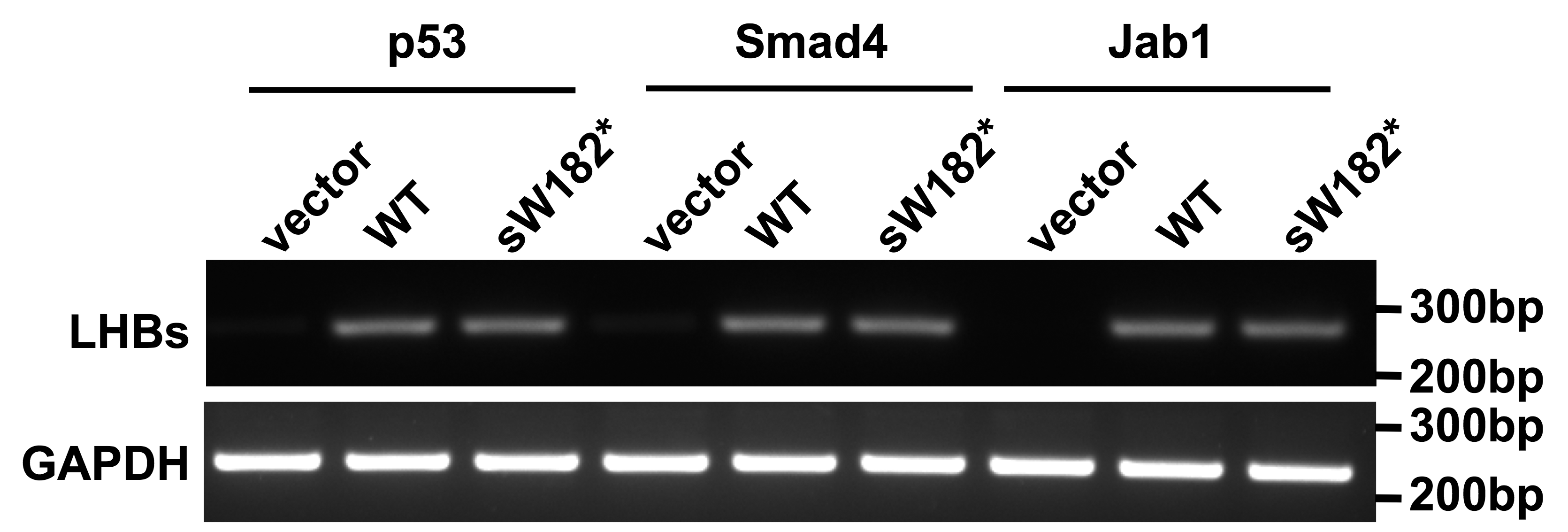

Supplement: S1 Fig — Huh-7 cells were cotransfected with p53, Smad4 or Jab1. The expression of the LHBs mRNA was confirmed by reverse transcription (RT)-PCR. GAPDH was used as a control. (TIFF) [file pone.0208665.s001.tiff]
